# Barriers and facilitators related to use of prenatal care by inner-city women: perceptions of health care providers

**DOI:** 10.1186/s12884-015-0431-5

**Published:** 2015-01-16

**Authors:** Maureen I Heaman, Wendy Sword, Lawrence Elliott, Michael Moffatt, Michael E Helewa, Heather Morris, Patricia Gregory, Lynda Tjaden, Catherine Cook

**Affiliations:** College of Nursing Faculty of Health Sciences, University of Manitoba, 89 Curry Place, Winnipeg, MB R3T 2N2 Canada; Department of Community Health Sciences, College of Medicine, Faculty of Health Sciences, University of Manitoba, Winnipeg, MB R3E 0W3 Canada; Department of Obstetrics, Gynecology & Reproductive Sciences, College of Medicine, Faculty of Health Sciences, University of Manitoba, Winnipeg, MB R3E 0L8 Canada; School of Nursing and Department of Clinical Epidemiology and Biostatistics, Faculty of Health Sciences, McMaster University, Hamilton, ON L8N 3Z5 Canada; Department of Medical Microbiology, College of Medicine, Faculty of Health Sciences, University of Manitoba, Winnipeg, MB R3E 0J9 Canada; Department of Pediatrics and Child Health, College of Medicine, Faculty of Health Sciences, University of Manitoba, Winnipeg, MB R3A 1S1 Canada; Faculty of Nursing, University of Alberta, Edmonton, AB T5G 1C9 Canada; Department of Nursing, Red River College, Winnipeg, MB R3H 0J9 Canada; Public Health, Winnipeg Regional Health Authority, Winnipeg, MB R3A 0X7 Canada; Population and Aboriginal Health, Winnipeg Regional Health Authority, Winnipeg, MB R3B 1E2 Canada

**Keywords:** Prenatal care, Pregnancy, Health care providers, Provider perceptions, Barriers, Facilitators, Qualitative study

## Abstract

**Background:**

Socioeconomic disparities in the use of prenatal care (PNC) exist even where care is universally available and publicly funded. Few studies have sought the perspectives of health care providers to understand and address this problem. The purpose of this study was to elicit the experiential knowledge of PNC providers in inner-city Winnipeg, Canada regarding their perceptions of the barriers and facilitators to PNC for the clients they serve and their suggestions on how PNC services might be improved to reduce disparities in utilization.

**Methods:**

A descriptive exploratory qualitative design was used. Semi-structured interviews were conducted with 24 health care providers serving women in inner-city neighborhoods with high rates of inadequate PNC. Content analysis was used to code the interviews based on broad categories (barriers, facilitators, suggestions). Emerging themes and subthemes were then developed and revised through the use of comparative analysis.

**Results:**

Many of the barriers identified related to personal challenges faced by inner-city women (e.g., child care, transportation, addictions, lack of support). Other barriers related to aspects of service provision: caregiver qualities (lack of time, negative behaviors), health system barriers (shortage of providers), and program/service characteristics (distance, long waits, short visits). Suggestions to improve care mirrored the facilitators identified and included ideas to make PNC more accessible and convenient, and more responsive to the complex needs of this population.

**Conclusions:**

The broad scope of our findings reflects a socio-ecological approach to understanding the many determinants that influence whether or not inner-city women use PNC services. A shift to community-based PNC supported by a multidisciplinary team and expanded midwifery services has potential to address many of the barriers identified in our study.

**Electronic supplementary material:**

The online version of this article (doi:10.1186/s12884-015-0431-5) contains supplementary material, which is available to authorized users.

## Background

The Chief Public Health Officer’s Report on the State of Public Health in Canada emphasizes that “Ongoing prenatal care is important to achieving a healthy pregnancy and birth, and positively influencing the health of the child in the early years. It provides a pregnant woman with the opportunity to access health information and identify risks and underlying factors that can influence her health and the health of her fetus/child” [1] (p. 52). In Canada, a variety of health care providers deliver primary prenatal care (PNC) services, most notably family physicians, obstetricians, midwives and nurse practitioners. In addition, primary care nurses, public health nurses, social workers, and nutritionists are often involved in PNC. A series of visits with a primary health care provider, beginning in the first trimester and continuing at recommended intervals throughout the pregnancy, constitutes the norm for PNC in Canada [2], although debate exists on the ideal frequency and content of PNC visits [3]. Women are defined as receiving inadequate PNC if they obtain less than the recommended number of visits, commence care after the first trimester, or receive no PNC at all [4].

Although PNC is universally available through Canada’s publicly funded health care system, disparities in utilization exist. Our previous research found wide variation in rates of inadequate PNC throughout the province of Manitoba from 1991 to 2000 [5]. In particular, rates of inadequate PNC ranged from 1.1% to 21.5% across 25 neighborhoods in the capital city of Winnipeg, and those neighborhoods with the highest rates were clustered in or near the inner-city [5]. These disparities have persisted over time, as reflected by rates of inadequate PNC from 2007/08 to 2008/09 reported in a recent provincial perinatal surveillance report [6].

A number of quantitative studies in the last two decades have investigated factors associated with inadequate PNC [7-22], including a recent systematic review of determinants of inadequate PNC in high-income countries [23]. Our previous research found that Manitoba women were more likely to receive inadequate PNC if they had low incomes, reported high levels of perceived stress, had low self-esteem or identified themselves as Aboriginal [24]. In the Canadian Maternity Experiences Survey, teenagers, women with lower levels of education, and those reporting lower incomes were found to be more likely to initiate PNC after the first trimester [25]. The same survey determined that Manitoba had the highest proportion of women who reported not getting PNC as early as they wanted (18.6%) and a high proportion of women who initiated PNC after the first trimester (7.8%), compared to other provinces [25], suggesting that health system factors related to the accessibility of care may also play a role.

Qualitative studies have also provided insight into barriers and facilitators associated with accessing PNC [26-32]. In a meta-synthesis of qualitative studies of barriers to PNC among marginalized women in high-income countries, Downe and colleagues [33] found that, in addition to factors such as recognition and acceptance of the pregnancy, women took into account the perceived gains and losses in obtaining care as they pertained to their personal resources (e.g., social support, time, money). Issues around delivery of services (e.g., cultural sensitivity, quality of care, respect, trustworthiness) also influenced women’s decisions about obtaining PNC.

To date, the majority of both quantitative and qualitative studies on PNC have been conducted in the United States, which differs from Canada not only in how health care is financed and delivered, but also in racial/ethnic composition. In addition, the majority of studies have focused on barriers and facilitators related to use of PNC from the perspective of women. Lacking in all of this research has been a dialogue with health care providers who provide PNC to women in Canada. Sword [34] argues that seeking the experiential knowledge of health care providers and administrators is important to illuminate the socio-political context of program and service delivery. Health care providers who work in inner-city environments are uniquely situated to offer perspectives on the broader determinants that affect their clients’ ability to access care, such as issues of poverty, poor housing and unemployment. In other research, health care providers have offered their perspectives on barriers to service use for postpartum depression [35], challenges and opportunities in caring for low-income pregnant adolescents [36], and the quality of PNC [37,38]. Only a few studies have investigated barriers or facilitators of PNC from the perspective of health care providers [20,39-43], and their suggestions on how best to improve access to PNC for inner-city women have rarely been sought.

In an effort to elicit the experiential knowledge of health care providers in inner-city Winnipeg, we set out to explore their perceptions of the barriers and facilitators to PNC for the clients they serve and their suggestions on how PNC services might be improved to reduce disparities in utilization. This work was part of a larger, mixed-methods study undertaken from 2007 to 2010 that also included a case–control study and a qualitative descriptive study with pregnant and postpartum inner-city women to inform health policy and practices related to PNC both locally and nationally. The case–control findings related to women’s assessment of barriers, motivators and facilitators of PNC utilization (N = 608) have been reported elsewhere [44].

## Methods

**Figure 1:**
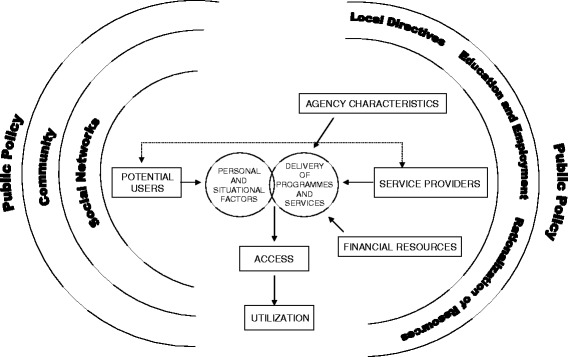
**A socio-ecological model of determinants of health services utilization.** Legend: Reproduced from Sword W: A socio-ecological approach to understanding barriers to prenatal care for women of low income. *J Adv Nurs* 1999, 29: 1170–1177.

A descriptive exploratory design guided the qualitative portion of the larger study. Such designs involve a comprehensive summary of events and the meanings participants ascribe to those events, a particularly useful approach when seeking answers to questions important to policy makers and practitioners [45]. Sword’s [34] socio-ecological model of determinants of health services utilization (Figure 1) provided a conceptual framework for the study. This model emphasizes the importance of two interacting systems: health services characteristics and an individual’s personal and situational factors.

The study was approved by the Education/Nursing Research Ethics Board at the University of Manitoba and the Assembly of Manitoba Chiefs Health Information Research Governance Committee, and permission to recruit participants was received from the Winnipeg Regional Health Authority (WRHA) Research Review Committee.

### Sampling and recruitment

We employed purposeful criterion sampling to obtain information-rich data [46] from health care professionals who provide PNC to women from eight neighborhoods located in Winnipeg’s inner city. These neighborhoods were chosen because of their high rates of inadequate PNC, as determined in the study mentioned above [5]. Health care providers were considered eligible to participate if they had a minimum of 2 years’ experience delivering PNC to inner-city women. Additionally, maximum variation sampling was used to garner a wide range of perspectives (e.g., various professional groups and settings, years of clinical experience, neighborhoods served) to facilitate a deeper understanding of PNC utilization [47]. Recruitment occurred through a variety of health care sites: private physician offices, the outpatient department of a tertiary care hospital, primary care community clinics, the midwifery program, and public health offices. Potential participants were offered a letter of invitation and, if interested in participating, were asked to contact the project coordinator to set up an interview time. Recruitment continued to the point of data saturation [48].

### Data collection

Data were collected using semi-structured, face-to-face interviews. These interviews took place during office hours in a private room at the workplace of each participant, who granted signed informed consent prior to the interview. On average, each interview lasted 40 minutes. The interviews were conducted by either the project coordinator or a graduate student, who both received training in interviewing skills and used the same interview guide to maintain consistency. The interview guide (see Additional file 1) was based on the socio-ecological model [34], and PNC was broadly defined for participants as “visits to a doctor, midwife, or nurse practitioner, as well as community-based programs and services, such as prenatal classes and public health nurse visits.” Each interview concluded with a brief demographic questionnaire. The interviews were audio-recorded and the recordings were transcribed verbatim by a professional transcriptionist. The interviewer then compared the transcript to the audio-taped interview to ensure accuracy.

### Data analysis

Each interview was coded using the process of content analysis [49,50]. The project coordinator worked with the first two authors of this paper to develop a preliminary coding scheme after reading the first few transcripts in their entirety. Within three broad topic areas (barriers, facilitators and suggestions), themes and subthemes were developed inductively and revised through the use of comparative analysis [48,51]. Throughout the analysis, variations and contradictions in the data were investigated to further understand the emerging themes [51,52]. The qualitative analysis software QSR NVivo Version 9 was used to assist with data analysis and organization of emerging themes and subthemes. Data were verified through conscientious adherence to research design, sampling to saturation, and methodological coherence [53]. Validation occurred by means of an audit trail (e.g., notes made on discussions and decisions pertaining to analysis), the use of NVivo for analysis, and promotion of inter-rater reliability through discussions among the project coordinator and the first two authors during the analysis phase [53,54].

## Results

A total of 24 participants were recruited into the study and represented a diverse group of health care providers who worked with inner-city pregnant women in Winnipeg: obstetricians (n = 7; 29%), public health nurses (n = 5; 21%), family physicians (n = 3; 13%), midwives (n = 2; 8%), hospital clinic nurses (n = 2; 8%), community clinic nurses (n = 2; 8%), nurse practitioners (n = 2; 8%) and a paraprofessional home visitor from the Families First program (n = 1; 4%). Participants worked primarily full time (n = 20; 83%) and reported a mean of 13.3 years of experience providing PNC in the inner-city (range 2–28 years). An attempt was made to sample both genders; however, the vast majority of those who agreed to be interviewed were female (n = 22; 92%), primarily due to the preponderance of females in some of the professions.

Health care providers identified a wide variety of barriers and facilitators to PNC for inner-city women in Winnipeg, and provided numerous suggestions for improving access and care. Table 1 summarizes the themes and subthemes in the topic areas of barriers, facilitators and suggestions.

**Table 1:** **Barriers and facilitators related to Use of Prenatal Care (PNC) and suggestions to improve use of PNC by inner-city women: perceptions of health care providers**

**Topic area**	**Themes and subthemes**
***Barriers***	***Caregiver qualities***
	• Too busy/lack of time
	• Negative personality characteristics (e.g., rude, judgmental)
	***Health care system barriers***
	• Lack of public awareness of PNC services
	• Shortage of health care providers who provide PNC
	***Personal barriers***
	• Logistical difficulties related to transportation and child care
	• Financial problems
	• PNC not viewed as a priority, no interest, not seen as important
	• Previous negative experience with/distrust of health care system
	• Personal pressures (e.g., addictions, intimate partner violence)
	• Lack of social support
	***Program and service characteristics: Inaccessible and/or inconvenient***
	• Geographic distance
	• Lengthy office wait
	• Short visits; rushed appointments
	• Inflexible or inconvenient hours
***Facilitators***	***Caregiver qualities***
	• Investing in relationship with client
	• Making women feel respected and valued
	• Effective communication skills
	***Caregiver approaches to provision of PNC***
	• Providing individualized, culturally sensitive care
	• Sharing health information with women, answering questions
	• Taking time with clients
	• Helping women understand importance of PNC
	***Multidisciplinary approach to PNC***
	• Referring women to additional services or programs
	• Using a team approach to meet women’s needs
	***Program and service characteristics***
	• Geographic proximity
	• Flexible hours/scheduling
	• Self-referral options for clients
	• Appointment reminders and follow-up contact
	• Expanding community-based clinics
	• Assistance with transportation and child care
	• Tangible rewards
***Suggestions***	***Make PNC more accessible and convenient***
	• Establish more community-based PNC clinics
	• Ensure closer proximity of PNC
	• Provide flexible hours/scheduling
	• Create drop-in access to PNC
	• Assist with transportation and child care
	***Motivate women to attend PNC***
	• Increase public awareness of PNC
	• Provide client-focused care
	• Explain rationale for PNC during visits
	• Offer tangible rewards
	***Make PNC more responsive to complex needs***
	• Maintain or enhance Health Baby and Families First programs
	• Offer PNC services specific for teens
	• Provide substance abuse support for pregnant women
	• Expand and promote midwifery services
	• Establish “one-stop shops” within a multidisciplinary environment

### Barriers to prenatal care

Barriers were defined as factors that make access to or use of PNC difficult or impossible for women. They were grouped into four themes: caregiver qualities, health care system barriers, personal barriers, and program and service characteristics.

#### Caregiver qualities

Many providers spoke at length about qualities in themselves or other care providers that posed a barrier to inner-city women, with most participants conceding that providers’ busyness or lack of time could be problematic for their clients. One obstetrician expressed an understanding of the situation in the following way:

*When we see them, because we are so busy … we depend a lot on … either the patient being autonomous, learning on their own or attending prenatal classes, or those kinds of things. So I would guess that somebody who doesn’t have the resources or the time and doesn’t have the ability to get their prenatal education from other places, it would be pretty unrewarding to come and see a busy obstetrician who comes in and says, “Hi, how are you? Is your baby moving?,” check your blood pressure, measure your tummy, and out you go. Because there is no relevance to them [inner-city women] in that model.*

Providers also noted that negative personality characteristics in health care professionals sometimes presented a barrier to care. Rude or judgmental caregivers in particular were seen as being potentially problematic for inner-city women seeking PNC. A general sentiment was that women have the potential to remember, for many years, the unkind actions and words from interactions with certain health care providers. One obstetrician noted:

*They usually won’t go back if they have had an experience where they felt that they haven’t been treated with respect … We have to be so careful to care for them within the context of what they can actually accomplish.*

#### Health care system barriers

Barriers also were identified within the health care system itself. A lack of public awareness of PNC services frequently was noted as a factor in inadequate PNC. A number of obstetricians commented that sometimes women are unaware that they could access their services without a referral, while other health care providers voiced the concern that women simply do not know where to go for PNC or how to access certain services. One community-based nurse remarked:

*And there is not really the dollars in health care to be doing a lot of advertising… about if you are pregnant come here for care, and most of …the clinics are busy just keeping up with their day-to-day stuff that everyone is a little bit hesitant to do a lot of advertisement in terms of encouraging people to come in, but if you don’t do any of that, then for the younger population who is getting pregnant who may not have had any contact with the health care system, they don’t really know where to go or how to get in for care.*

In addition, a shortage of health care providers who offer PNC was identified as contributing to poor access to PNC for inner-city women. A hospital-based nurse commented on how women without a health care provider often present to the obstetrical triage unit to obtain PNC, and provided the following explanation:

*There is such a limited number of physicians around so they [pregnant women] cannot get access to a family doctor. The obstetricians are really bombarded and loaded with admissions and the midwives cannot accept more [clients]. … but the thing is when some shy person calls the office and the receptionist says ‘no, we do not accept new people without a referral’, well …, if you can’t even get in, how can you get care?*

#### Personal barriers

Health care providers discussed the fact that a number of the women they see are dealing with personal barriers that may interfere with attending PNC appointments. The majority identified logistical difficulties related to transportation and child care. One of the midwives considered how these challenges may outweigh the value of PNC visits for many inner-city women:

*If you have to take your stroller and your three kids and take a bus exchange … the benefit is not worth the problems that it creates.*

Financial problems were identified as contributing to these logistical difficulties, with half the participants specifying a lack of money for transportation as a factor in their clients’ decisions not to attend PNC. Other providers commented on how women working in low paying jobs were reluctant to take time off work to attend PNC appointments.

Health care providers also commented that they believed PNC was not a priority for some women because they were not interested in PNC, did not think they needed it, or did not understand its importance. A family physician expressed thoughts about why some multiparous women did not seek PNC:

*For some of the moms who have multiple children, … I think they are of the minds that they have ‘been there, done that’ and that not much goes on at a prenatal visit, so therefore it doesn’t matter too much if I don’t go in. But I think it is important that they are aware that new issues can arise in any pregnancy.*

Women were also thought to avoid PNC because of a distrust of the health care system, sometimes attributed to past involvement with Child and Family Services. A negative past experience with the health care system or a provider was identified as an additional factor that potentially contributed to inadequate use of PNC services. An obstetrician explained the impact of a bad experience:

*I think it has a huge influence, if they have had a bad experience with a provider they’ll either say ‘well I don’t need them’ or ‘they didn’t do anything for me anyway’ or they will wait or they switch people [providers] and then you don’t have their whole history and stuff like that.*

Additional issues revolved around the personal pressures experienced by many inner-city women, who were described as having multiple crises in their lives or as “just being busy with surviving”. The most frequently mentioned personal pressures included addictions, intimate partner violence, and socioeconomic issues such as poverty (e.g., “money problems, lots of debt”), food insecurity, and housing problems or a transient lifestyle. One community clinic nurse summed up a number of these personal pressures and their perceived impact on women’s decisions to attend PNC as follows:

*But if you don’t really have a need [to go for PNC], if you are feeling great and you haven’t had any spotting and are not that nauseated, everything is kind of going along … you need to be at a fairly secure place in your life in terms of housing, shelter, stable relationships, stable job to … have the luxury of … health promotion pieces. You know, if you are phoning the food bank every week to find out when you can go in for extra food, you are at the school a couple of times a week with issues with your other kids … if you are involved with CFS [Child and Family Services], if you have anybody in the family with another health problem and you are helping them get to doctors, … worrying about your own health is low, low down on the list, particularly if you feel okay.*

Often these personal pressures were thought to be exacerbated by a general lack of social support to assist women to deal with these issues. An obstetrician commented that many of the women “don’t have a reliable partner, or they have an abusive partner, which is worse.” Other providers commented on the isolation experienced by First Nations women who relocated to the city from their northern reserve: “a lot of them are single parents and their parents or grandparents …are on reserves, so they don’t have access to extended family who could …help them out.”

#### Program and service characteristics

Barriers related to program and service characteristics also emerged, particularly those pertaining to inaccessible or inconvenient PNC services. Health providers admitted that, for some women, services are simply too far away, visits are often too short, clinic hours may be inflexible or inconvenient, and the amount of time spent waiting in the office is too long. One obstetrician put it this way:

*They often have to bring the kids with them and if they … have to wait an hour in the waiting room with these two or three rambunctious, mewling and crying kids,… to see me, all for a grand total of a 5-minute visit, that is a real disincentive for women to come in.*

High-volume practices that resulted in rushed PNC appointments were identified as an additional barrier. As another obstetrician remarked:

*Some of the care providers … are interested in a high-volume practice rather than a high-quality practice, so that people who attend prenatal care … sometimes just feel like cattle in a stampede you know. …That discourages people from attending their next appointment.*

### Facilitators of prenatal care

Facilitators were defined as external factors that make access to PNC easier for women and included the following themes: caregiver qualities, caregiver approaches to provision of PNC, a multidisciplinary approach to PNC, and program and service characteristics.

#### Caregiver qualities

Participants deemed certain personal characteristics or qualities in caregivers to be essential for facilitating the use of PNC among their inner-city clients. More than half of the providers commented on the importance of being willing to invest in a relationship with the client, and the need to “care about them in their life.” One obstetrician discussed the importance of building rapport with the woman:

*I think that a certain amount of prenatal care is important. It is important to have a few basic tests done. Do we need to see people as often as the book tells us to? No. But to me the purpose of that is to build the relationship and the rapport to help with some education and understanding. And give people a reason to maybe keep coming back.*

Care providers also spoke in general terms about the importance of making women feel respected and valued. For example, one obstetrician commented:

*This is probably going to sound kind of crazy, but it [the PNC environment] has to help them celebrate the pregnancy and value the child, and … they need to have a place where they are going to feel welcome and respected and valued, and they do come if you give them that feeling.*

Additionally, participants often identified the need for health care providers to have effective communication skills, including listening, showing interest, and being non-judgmental and accepting.

#### Caregiver approaches to provision of PNC

When asked to identify factors that facilitated access to PNC, health care providers tended to discuss approaches to provision of PNC that they themselves had carried out. Many of the participants discussed the importance of providing individualized or contextually-based care when working with inner-city women. Such care allowed providers to connect and develop a relationship with their clients based on mutual trust and respect. An obstetrician working in this area for many years stated:

*We have to be so careful to care for them within the context of what they can actually accomplish … We make a lot of compromises in care to keep them coming, so we don’t worry about quitting smoking, we worry about smoking less.*

Other providers emphasized the need to provide culturally sensitive care as part of individualized care, particularly for Aboriginal women and newcomers to Canada. A nurse who worked in a hospital outpatient clinic commented:

*I think just keeping in mind that …we have a diverse population [in the inner-city]. So we have to be very focused on …individualizing our care… to keep in mind the different languages, the different cultures… I think we have to have interpreters available and people who make it as comfortable for our clients as possible.*

A number of health care providers discussed the benefits of the educational elements of PNC, particularly in an environment where appointments are not rushed and women have sufficient time to ask questions and get information. Many participants emphasized the importance of taking time with clients, as reflected in the following comment made by a midwife:

*Women want time. They want to be able to talk about what they are doing, and for women who aren’t educated, don’t know the right questions, or how to say things, it often takes a lot of time just sitting with them to open to the point where they will talk about a bad discharge smell or… the baby hasn’t been moving for the last two days … It really talks to having enough time to get to know the woman and for them to feel like they are welcomed and they are listened to and they are not hurried out.*

Providers also noted their own role and responsibility in “selling” women on the importance of PNC, in addition to sharing information with clients. One obstetrician commented:

*I think women aren’t going to come for an appointment if they don’t perceive a benefit in it. So it needs to be relevant to them and they need to understand what the point is … If they understand that the point of them coming in is so that we can make sure that the baby is growing well and their blood pressure is okay … I think that would be a big benefit of the education side of it.*

#### Multidisciplinary approach to PNC

Both community-based providers and those working in tertiary care hospital facilities discussed the benefits of a multidisciplinary approach to PNC as an efficient and effective way to meet women’s needs and encourage them to attend care. The providers discussed their working relationships with social workers, mental health providers, dieticians, public health nurses, primary care nurses, physicians, and, in one case, a traditional Aboriginal healer. Many spoke about their role in referring women to additional services or programs to supplement present care or in receiving referrals from other services. Many of the providers appreciated a team approach in addressing client issues, and women were viewed as being more likely to attend PNC visits if they could get their other needs met at the same time. One family physician offered this perspective:

*I think a lot of the limitations are getting people [pregnant women] here [to the clinic] and ensuring that they get here regularly, but once they are here, I think being in a very multidisciplinary practice … we do a pretty good job of meeting those needs when they are here… we do have both physicians and a nurse practitioner as well as a primary care nurse who …has a lot of background in obstetrical care, so that we have got those resources to provide education and support …. We also have some of the mental health resources on site … which we do end up connecting quite a few of our prenatal clients with.*

Participants often mentioned the benefits of connecting pregnant women to the programs provided by the provincial government as part of its Healthy Child Manitoba initiative [55]: the Healthy Baby Community Support Programs offer social support and informal learning opportunities on a drop-in basis to encourage early, regular prenatal care and promote healthy infant development; the Healthy Baby Prenatal Benefit is a monthly income supplement available to eligible low-income women to help them eat well during pregnancy; and the Families First program offers home visiting supports to families from pregnancy to school entry assessed as living in conditions of risk [56].

#### Program and service characteristics

Health care providers identified a number of program and service characteristics that facilitated PNC for inner-city women. Providers felt strongly that service-related characteristics such as geographic proximity to where women live, flexible hours, ease of scheduling of appointments, and self-referral options for clients were critical in making PNC services more accessible and convenient for their clients. Some of these characteristics were common to PNC provided at community-based clinics. Providers also noted that appointment reminders and follow-up contact were important for women who missed appointments.

Several providers spoke about the benefits of assisting women with transportation to attend PNC visits, such as through the provision of bus tickets or taxi slips. Some providers even went so far as to provide transportation, such as a community clinic nurse who spoke about her unsanctioned role in ensuring street-involved women get to their PNC appointments:

*I have picked them up and driven them to Dr. X for about five or six pregnancies now. But that is the only way they will go is if I take them. So … that is really something that we aren’t supposed to do … but I know that is the only way that they will get there. And one of them is HIV-positive so she has lots of health needs and really needs to get there so I drive her.*

Many of the providers also discussed the value of providing assistance with child care, and described examples of on-site child care services or family-friendly environments where clients’ children could be supervised by family members or friends. However, one family physician offered the following insight:

*I would like to say having child care on-site would help but … I think one of the roadblocks is actually bringing all the kids in with them, so I think it is a matter of having support in the home … to allow them to come in for an appointment.*

Offering tangible rewards to women (e.g., food, prenatal vitamins, milk coupons, clothing) was another service-related feature that providers deemed important to facilitate attendance at PNC, although not all PNC sites had funding available for this purpose. Most respondents referred to the success of incentives offered by the Healthy Baby programs. As a public health nurse noted:

*The sites offer vitamin D, they have milk coupons and they have food coupons now, like for frozen vegetables and orange juice, and so that for sure is a draw [to attend].*

### Suggestions to improve prenatal care

Health care providers were invited to discuss ways to reduce disparities in the use of PNC services. They offered a wide variety of suggestions that fell under three themes: make PNC more accessible and convenient for inner-city women, motivate women to attend PNC, and make PNC more responsive to women’s complex needs. Not surprisingly, many of the suggestions mirror the facilitators discussed above.

#### Make PNC more accessible and convenient

The most common suggestion to make PNC more accessible and convenient, made by more than half of participants, was to establish a greater number of community-based PNC clinics located closer to where women live. More specifically, participants wanted to see PNC offered at inner-city community clinics, schools, Healthy Baby sites, organizations serving street-involved women, and store-front clinics. One family physician summed it up this way:

*I think the care should go out into the community if at all possible … be decentralized … So we either take the care out there or we help some of the clinics develop antenatal care systems within their primary care systems.*

One obstetrician reflected on this topic and offered the following suggestion:

*Ideally, it would be nice to set up some sort of, even just basic prenatal clinic… in the inner-city area where it would be a catch-all place for people to go so that if they don’t have a doc [doctor], it is a known public place for prenatal care. And whether it is run by nurse practitioners, that would be even great and then they could refer on to the obstetricians.*

Flexible scheduling, such as evening and weekend clinic hours, and establishing drop-in PNC services also were frequently suggested as strategies to improve access.

Many participants suggested that PNC providers could assist with transportation by offering bus tickets, bus passes, or taxi vouchers or by running a van to bring women to the clinic. One obstetrician recommended that transportation support should be routinely offered to inner-city women:

*It is really hard to get a bus pass out of Child and Family Services or Social Assistance. It should just be automatic that they [women] get a bus pass when they are pregnant. And for whomever they have to bring with them too. I think transportation is a big issue.*

Participants also offered specific ideas to assist with child care, such as having additional space, play areas, supervised child minding on site, and home respite services.

Providers also emphasized the need to promote public awareness of where PNC services are located to improve access to care and reduce disparities in usage. A family physician said:

*We are taking on a lot of new clients now and one of our high risk groups that we have identified is … pregnant women - that those will be taken right away above our waiting list. But a lot of people don’t know that this place [community-based clinic] exists or what resources are out there, so they don’t know where to go. I think having ways of having that information more available to them … to know where they can access a nurse practitioner or a family doc [doctor].*

A public health nurse offered some innovative suggestions:

*We need to find ways to get more information about [PNC] out there and if you want to reach those people [pregnant women], you go to where they are at. They are at food banks, so I think we should be at food banks and tap in to that…. And even if something [information about PNC] was to be … [included with] their cheques for income assistance.*

#### Motivate women to attend PNC

Providers frequently recommended promoting public awareness of the importance of PNC in order to help motivate women to get PNC. A public health nurse offered the following suggestion:

*If we could convince them [girls and young women] somehow that prenatal care is important for them and their baby … whether that’s through things we do in the school setting …[or] through the media like TV ads… that kind of promotes getting early prenatal care and regular prenatal care … if we kind of make it really public and it becomes part of what they grow up learning.*

Providers also felt they had a responsibility to incorporate a client-focused approach into their practice, as well as to explain the rationale for attending PNC visits to their clients. A family physician discussed the importance of motivating women:

*You have to be connecting with these women one on one and getting them in and giving them a reason to come in … for appointments and give them a reason to want to take care of themselves.*

Similarly, a community educator stressed the need to better understand why women forego PNC:

*So the question is, for the ones that aren’t coming in, why aren’t they coming in? …What can we do to … encourage them or to make that easier? … Partly it might be we really need to be able to sell prenatal care to them.*

Lastly, a number of health care providers wanted to see more tangible rewards or incentives offered for attending PNC (e.g., food, money, clothes, diapers, prenatal vitamins, milk coupons).

#### Make PNC more responsive to women’s complex needs

Specific program-related suggestions to make PNC more responsive to the complex needs of inner-city women included maintaining or enhancing the province’s Healthy Baby and Families First programs, providing PNC support specifically designed for teens, and improving access to substance abuse programs for pregnant women. An obstetrician stated:

*I would really like to have a substance abuse professional available for easy consultation. Because for women who have [addiction] issues during pregnancy, there isn’t an easily available way to get them help. There are waiting lists for everything and sometimes when a person decides this is the day that they want to kick the habit, today is the day that we should be able to provide something, even if it is just somebody to talk to them.*

Many providers felt that expanding and promoting midwifery services would improve the use of PNC among inner-city women. As one midwife stated, “I really do see a huge benefit for marginalized, traditionally under-served populations with midwifery care*”*, while a public health nurse elaborated on reasons why midwifery would be beneficial:

*They [midwives] have the time to spend. They can involve siblings if there are some. They have a holistic view of birth and aren’t just looking for problems; they are looking at birth as normal and that is not a medical condition sort of thing. They are very non-judgmental … they can be there for the whole labor with this woman and I think that is huge for these women. A lot of them [inner-city women] have been sexually abused and …that plays a role in terms of labour and birth so to have that familiar face. A lot of them [women] don’t have supports,… so to have the midwives, I think it is good.*

A number of health care providers also suggested more widespread use of a “one-stop shop” approach, in which PNC would be offered within a multidisciplinary environment with various types of care providers working under the same roof. One obstetrician said this would create “the best bang for your buck” while a nurse practitioner described how this approach would address complex needs:

*I would like to see [high-risk] prenatal patients walk into a setting where they would … have access to a social worker or a counselor or a dietician and a nurse practitioner all in the same setting … Sometimes … you only have that one time to catch them and sometimes you want to do as much as you can … It would be nice if there was a way that we could provide that for the patients when … they need it.*

## Discussion

Health care providers in our study identified a myriad of barriers, facilitators, and suggestions to reduce disparities in the use of PNC in Winnipeg. Many were related to the personal and social situations faced by inner-city women, while others pertained to how services are provided, both at the level of individual caregivers and programs and within the broader health care system. The broad scope of our findings reflects Sword’s [34] socio-ecological model for understanding the many types of factors that may influence whether or not low-income women use PNC services.

### Barriers

Health care providers in this study demonstrated awareness of an extensive array of barriers to PNC experienced by the inner-city women for whom they provided care. Many of the barriers identified in this study are congruent with those identified in Johnson and colleagues’ [43] study of 61 PNC providers who served low-income minority women in Washington, DC using a structured questionnaire. Barriers identified in both studies included personal barriers (e.g., family problems, intimate partner violence, lack of awareness of where to go for PNC, denial of the need for PNC, transportation problems, child care problems) and health care provider or system issues (e.g., negative staff attitudes, inconvenient clinic hours, long wait for appointment), reflecting the two interacting systems in Sword’s [34] model. Although barriers such as “no health insurance” and “no money to pay for PNC” were unique to the Washington health care setting, providers in Winnipeg frequently mentioned financial problems as impacting women’s access to care.

Our results also are similar to two other studies in the United Stated that explored barriers to PNC as perceived by both women and health care providers. Teagle and Brindis [20] surveyed adolescents and health care providers to compare their perceptions of barriers and facilitators to PNC. Adolescents discussed barriers such as financial problems, lack of transportation, and long wait times at appointments, while providers were more likely to speak to adolescents’ personal barriers (e.g., feeling depressed, difficulties at home). These authors attributed the differences in opinions between these two groups to poor levels of communication, suggesting that, congruent with our findings, this in itself may pose a barrier to PNC. Aved and colleagues [39] also explored barriers to PNC (and, to a lesser extent, suggestions to improve care) as identified by low-income women and health care providers. Women identified barriers such as providers not taking new patients, transportation problems and geographic distance. In the same study, a focus group with seven obstetricians tended to concentrate on the physicians’ own barriers to providing care to this population rather than their perceptions of low-income women’s experiences. In contrast, many providers in our study identified that low-income women often faced financial and logistical barriers to accessing care, suggesting that a lack of awareness of clients’ challenges was not a core problem.

In our study, providers discussed barriers related to program and service characteristics and how these might vary by practice setting (e.g., hospital outpatient clinic, community-based multidisciplinary clinic, midwifery practice). In the United States, Gilbert and colleagues [41] explored health care providers’ perceptions of how various health care settings influenced the use of prenatal risk-reduction services. They found significant differences between a large HMO practice and private practices, and concluded that the “practice setting strongly influenced providers’ behavior, and settings differed by continuity of care, availability of resources, and organized support for risk prevention” (p. 42).

Our findings were largely consistent with one other qualitative study that explored barriers to PNC among women enrolled in a Medicaid managed care plan in Tennessee [40]. Gazmararian and colleagues [40] conducted focus groups women and health care providers and found that both groups of respondents thought that problems with transportation, lack of knowledge about Medicaid managed care, and substance abuse were barriers to receiving PNC. Lack of education, problems with child care, and limited office hours were additional barriers mentioned by providers, while women in the study discussed negative treatment by office staff, lack of rapport with providers, and not knowing they were pregnant as barriers.

### Facilitators

Few studies have addressed facilitators related to use of PNC from the perspective of health care providers. Interestingly, many of the facilitators identified by participants in this study were similar to the findings of a qualitative study by members of this research team that explored health care providers’ and women’s perspectives of quality of PNC [38]. The themes related to quality of care included information sharing, women-centeredness, respectful attitude, approachable interaction style and taking time [38], all of which were identified as health services level facilitators of PNC in our current study. This suggests that provision of high quality interpersonal care processes may promote involvement of women in their own care, and keep women engaged in care.

Health care providers in our study also spoke about their own roles in facilitating service delivery by, for example, offering culturally sensitive care. Smith and colleagues [42] reported on interviews and small group discussions held with stakeholders, some of whom were health care providers, related to caring for pregnant and parenting Aboriginal women in Canadian rural and urban communities. The study found that, for this population, emotional safety was critical to accessing PNC, and many subthemes corresponded to PNC and provider qualities voiced by participants in our study (e.g., non-judgmental and respectful attitude, client-directed care, outreach and visibility). The authors also concluded that “… the intention of care must be situated within a broader view of colonizing relations to improve early access to, and relevance of, care during pregnancy and parenting for Aboriginal people” [42] (p. E27). This point is particularly relevant to the current context as Winnipeg has the largest Aboriginal population of all Canadian capital cities [57] and the eight neighborhoods in our study include a high proportion of Aboriginal women [44].

In addition, many of our participants felt that making PNC services more accessible and convenient (e.g., by providing support for child care and transportation and offering services close to where women live) would help inner-city women attend care. Incentives were also identified as a facilitator of PNC, similar to the finding of Aved and colleagues [39], who reported that “although some of the physicians were opposed to the idea of patient incentives, others suggested that financial or other incentives (particularly related to transportation and child care assistance) would improve compliance rates” (p. 497).

### Suggestions to improve prenatal care

Health care providers’ ideas about how to improve utilization of PNC are largely lacking in previous studies. Our study was designed to address this knowledge gap in the belief that providers could contribute rich information informed by their experiences within the health system and their daily interactions with the pregnant women they serve. Health care providers made more than 80 different suggestions of how PNC services might be improved for inner-city women, with those most often identified forming the themes and subthemes reported in the findings.

The most frequent suggestion was to establish more community-based PNC services. Currently, PNC services for inner-city women in Winnipeg are located in tertiary care hospitals, private obstetrician offices in the inner-city area and elsewhere, and a small number of community health clinics/offices staffed by family physicians or nurse practitioners. Registered midwives also provide PNC in Winnipeg, but a shortage of midwives exists due, in part, to limited training availability and an insufficient number of funded positions in the province. A shift to community-based care supported by expanded midwifery services has the potential to address many of the barriers identified in our study related to program and service characteristics (geographic distance, long waits for short visits), caregiver qualities (lack of time), and women’s personal barriers (travel difficulties, child care).

### Strengths and limitations

A major strength of this study is its focus on exploration of barriers and facilitators of PNC from the perspective of a diverse group of health care providers. The sample size of 24 providers ensured that we captured a broad range of perspectives and data saturation was achieved. Strategies were put in place to ensure rigor of the analytic process and hence validity of the study findings. Study limitations relate mainly to the sample in that the majority of care providers were female, and therefore the views of male health care providers may be under-represented. There was an over-representation of midwives and an under-representation of obstetricians in the sample relative to the proportion of these caregivers in the population. Health care providers interviewed for this study spoke in relation to their experiences of caring for inner-city women in Winnipeg, Manitoba. The findings will have varying degrees of transferability to other settings.

## Conclusions

The broad scope of our findings reflects a socio-ecological approach to understanding the many determinants that influence whether or not inner-city women use PNC services. This is the first study to document the views of Canadian health care providers with respect to PNC for inner-city women. The barriers and facilitators they identified have led to a number of recommendations that will inform changes in practice and policy to improve the use of PNC services in this population. Future research needs to focus on implementation and evaluation of new models of care that incorporate these suggestions to optimize the health of inner-city pregnant women and their children.

## Additional file

Additional file 1:
**Interview Guide: Health Care Providers.** Description of data: Interview guide used with health care providers in the qualitative component of the study “Factors associated with inadequate prenatal care among inner-city women in Winnipeg”, Principal Investigator: Dr. Maureen Heaman, College of Nursing, Faculty of Health Sciences, University of Manitoba.

## Abbreviations

MB: Manitoba
PNC: Prenatal care
N: Sample size
WRHA: Winnipeg Regional Health Authority
n: Number of participants
%: Percentage
HMO: Health Maintenance Organization
