# Supplementary material for: Barriers and facilitators related to use of prenatal care by inner-city women: perceptions of health care providers
Source: BMC Pregnancy Childbirth. 2015 Jan 16;15:2. doi: 10.1186/s12884-015-0431-5 (PMC4302607; doi:10.1186/s12884-015-0431-5)
Supplement: Additional file 1: — Interview Guide: Health Care Providers. Description of data: Interview guide used with health care providers in the qualitative component of the study “Factors associated with inadequate prenatal care among inner-city women in Winnipeg”, Principal Investigator: Dr. Maureen Heaman, College of Nursing, Faculty of Health Sciences, University of Manitoba. [file 12884_2015_431_MOESM1_ESM.docx]

**Additional File 1:**

**Interview guide used with health care providers in the qualitative component of the study “Factors associated with inadequate prenatal care among inner-city women in Winnipeg,” Principal Investigator: Dr. Maureen Heaman, College of Nursing, Faculty of Health Sciences, University of Manitoba**

Prenatal care includes visits to a doctor, midwife, or nurse practitioner, as well as community-based programs and services, such as prenatal classes and public health nurse visits. We know that many factors play a role in determining whether inner-city women use prenatal care or not. In this interview, I would like to talk to you about these factors.

I would like to begin by asking some questions about the prenatal care services you provide (or administer).

1. Tell me about the prenatal care services or program you provide (or administer).

- Probe for description of the service, location, characteristics of clients, proportion of clients from inner-city, etc.

2. Are there aspects of prenatal care that you would like to provide but can’t?

- Probe for constraints on provision of care (i.e., policies, financial resources, agency characteristics, availability of service providers, characteristics of potential users)

3. What other prenatal services or programs do women in your area commonly use?

- Probe re: physician or midwife visits, prenatal classes, Healthy Baby program, Healthy Start program, Families First program, Internet resources, etc.

I would like to go on and explore things that make it easy or difficult for women to use these

services and programs.

4. What, if anything, makes it easy for inner-city women to use your service or program?

- Probe re: ease of making appointments, waiting time, location, hours of service, attitudes of care providers, continuity of care, costs, childcare, transportation, support from family and friends, characteristics of the neighborhood, etc.

5. What, if anything, makes it difficult for inner-city women to use your service or program?

- Probe re: ease of making appointments, waiting time, location, hours of service, attitudes of care providers, continuity of care, costs, childcare, transportation, lack of support, characteristics of the neighborhood, etc.

6. What do you think are the perceived needs and expectations of women regarding prenatal care services and programs? How well do current services meet these needs and expectations?

7. How much of an influence do you think past experiences with doctors, nurses and other health care providers play in how readily people are likely to use prenatal services and programs?

- Probe re: perceived quality of care received, nature of the relationships, perceived benefits, negative aspects

Finally, I would like to hear from you about what other prenatal services and programs you

would like to see in your community.

10. How could current prenatal services and programs be improved to better meet the needs of women in your community?

11. Are there services or programs for pregnant women that you wish were available in your community but aren’t?

Thank you for taking the time to talk with me. You have shared a lot of information that will help us better understand women’s needs during pregnancy and how these can best be met. Is there anything else you would like to add?
